# The added value of diffusion-weighted imaging in the preoperative assessment of endometrial cancer

**DOI:** 10.1590/0100-3984.2018.0054

**Published:** 2019

**Authors:** Rui Tiago Gil, Teresa Margarida Cunha, Mariana Horta, Ines Alves

**Affiliations:** 1 Serviço de Radiologia, Instituto Português de Oncologia de Lisboa Francisco Gentil, Lisboa, Portugal.; 2 Serviço de Radiologia, Hospital. Dr. Nelio Mendonça, Funchal, Portugal.

**Keywords:** Endometrial neoplasms, Magnetic resonance imaging, Gynecology, Neoplasms, Endometrium/pathology, Neoplasias do endométrio, Ressonância magnética, Ginecologia, Neoplasias, Endométrio/patologia

## Abstract

**Objective:**

To evaluate the added value of diffusion-weighted imaging (DWI) in the preoperative assessment of myometrial invasion in endometrial cancer, in comparison with T2-weighted imaging (T2WI) and dynamic contrast-enhanced magnetic resonance imaging (DCE-MRI).

**Materials and Methods:**

This was a retrospective study involving 44 women with endometrial cancer who underwent preoperative 1.5 T MRI. Two radiologists, both of whom were blinded to the histopathology reports, performed a consensus interpretation of the depth of myometrial invasion and of the stage of the cancer, considering three sets of sequences: T2WI, DCE-MRI+T2WI, and DWI+T2WI. Accuracy, sensitivity, specificity, positive predictive value, and negative predictive value were calculated for each set. The accuracy was compared with *p*-value adjustment by the Benjamini-Hochberg procedure.

**Results:**

Among the 44 patients evaluated, DWI+T2WI demonstrated better diagnostic performance in assessing deep myometrial invasion and correctly staged more patients (n = 41) than did DCE-MRI+T2WI (n = 34) and T2WI (n = 22). The superior diagnostic accuracy of DWI+T2WI was statistically significant in comparison with T2WI (*p* < 0.05) but not in comparison with DCE-MRI+T2WI (*p* > 0.05).

**Conclusion:**

The addition of DWI apparently improves the diagnostic accuracy of MRI in the preoperative assessment of the depth of myometrial invasion in endometrial cancer, which may be particularly helpful in patients for whom contrast agents are contraindicated.

## Abstract (pt)

**Objetivo:**

Determinar o valor adicional da difusão (DWI) em ressonância magnética (RM) na avaliação pré-operatória da invasão miometrial do câncer do endométrio, em comparação com sequências T2 e com o estudo dinâmico após contraste paramagnético (*dynamic contrast-enhanced* - DCE).

**Materiais e Métodos:**

Estudo retrospectivo incluindo 44 mulheres com câncer do endométrio que realizaram RM 1,5 T pré-operatória. Dois radiologistas desconhecendo a histopatologia final realizaram interpretação consensual sobre a profundidade de invasão miometrial e o estádio da doença considerando três conjuntos de sequências: T2, DCE+T2 e DWI+T2. Para cada conjunto foram calculadas acuidade, sensibilidade, especificidade, valor preditivo positivo e valor preditivo negativo. A acuidade diagnóstica foi comparada com ajuste do valor de *p* utilizando o procedimento Benjamini-Hochberg.

**Resultados:**

DWI+T2 apresentou melhor performance diagnóstica na avaliação da invasão miometrial e permitiu estadiar corretamente mais pacientes (41/44) do que DCE+T2 (34/44) e T2 (22/44). A superior acuidade diagnóstica da DWI+T2 foi estatisticamente significativa quando comparada com T2 (*p* < 0,05), mas não quando comparada com DCE+T2 (*p* > 0,05).

**Conclusão:**

A interpretação da DWI+T2 melhorou a acuidade diagnóstica da RM na avaliação pré-operatória da invasão miometrial do câncer de endométrio, o que poderá ser particularmente útil em pacientes com contraindicação para contraste paramagnético.

## INTRODUCTION

Endometrial cancer is the leading gynecological cancer in high-income countries and the sixth most common in women worldwide^(^^1^^,^^2^^)^. More than 90% of all cases of endometrial cancer occur in women > 50 years of age, whereas only 4% occur in women < 40 years of age^(^^2^^)^. Endometrial cancer is typically diagnosed at an early stage (80% of cases being diagnosed in stage I) during the investigation of atypical bleeding in postmenopausal women, with five-year survival rates of over 95% in such cases^(^^3^^)^. However, overall five-year survival varies widely, from 20% to 95%, the prognosis mainly depending on three factors^(^^4^^)^: histological subtype and grade; local tumor stage at diagnosis; and the presence or absence of lymph node metastases. Although the grade and histological subtype of endometrial cancer can be diagnosed through endometrial sampling, tumor staging is traditionally performed intraoperatively, according to the International Federation of Gynecology and Obstetrics (FIGO) guidelines, which include the use of total abdominal hysterectomy, bilateral salpingo-oophorectomy, peritoneal lavage, and pelvic/para-aortic lymphadenectomy, depending on the findings at intraoperative staging^(^^5^^)^.

The depth of myometrial invasion represents the morphological feature with the greatest prognostic value in endometrial cancer, correlating with tumor grade, lymph node metastases, and overall patient survival^(^^4^^,^^6^^)^. Tumor invasion to greater than 50% of the myometrial thickness translates to a six to seven times greater risk of pelvic and para-aortic lymph node metastases, and patients should be considered candidates for a more aggressive surgical approach^(^^7^^)^. However, the value of routine lymphadenectomy in early endometrial cancer remains controversial. Two large prospective studies, collectively including approximately 2000 women, demonstrated no benefit of pelvic lymphadenectomy in terms of overall and recurrence-free survival in women with early-stage (IA) preoperative endometrial cancer, in comparison with less invasive surgical approaches^(^^8^^,^^9^^)^. Accurate preoperative assessment of the depth of myometrial invasion and pathological staging is therefore crucial to the planning of the appropriate surgical approach^(^^10^^)^.

Magnetic resonance imaging (MRI) has proven to be the most effective technique in the preoperative evaluation of endometrial cancer, showing great accuracy in the assessment of myometrial invasion, cervical stromal invasion, and lymph node metastases^(^^11^^,^^12^^)^. Although MRI was not formally incorporated into the revised FIGO staging system for endometrial cancer, it is widely used to assess the stage of the disease and to plan the appropriate therapeutic approach^(^^4^^,^^8^^)^. The standard MRI protocol includes high-resolution T2-weighted imaging (T2WI), in various planes, and multiphase dynamic contrast-enhanced (DCE)-MRI^(^^13^^)^. However, there is no consensus regarding the best protocol, and recent studies have produced contradictory results, demonstrating no significant added value of DCE-MRI, either in the assessment of myometrial invasion or in the staging-nor have there been any reported differences between T2WI and DCE-MRI+T2WI in terms of interobserver agreement^(^^4^^,^^6^^)^.

Simultaneously to the technical improvements and growing general interest in the use of diffusion-weighted imaging (DWI) in the evaluation of the female pelvis, studies have obtained encouraging results with the use of DWI in the preoperative assessment of endometrial cancer^(^^11^^,^^13^^)^. However, further studies are needed in order to demonstrate its added value and to consolidate its use in clinical practice. DWI is a functional imaging technique that provides information about water mobility, tissue cellularity, and the integrity of cellular membranes^(^^14^^)^. On DWI, endometrial cancer demonstrates restricted diffusion in comparison with that of normal myometrial tissue, resulting in high signal intensity at high b-values (500-1000 s/mm^2^) and low apparent diffusion coefficient (ADC) values^(^^2^^,^^14^^)^. ADC values are also significantly lower in endometrial cancer than in normal endometrium or in benign conditions such as endometrial polyps, leiomyomas, and endometrial hyperplasia^(^^15^^,^^16^^)^.

The purpose of this study was to evaluate the added value of DWI (as an adjunct to T2WI) in the preoperative assessment of endometrial cancer. Our main hypothesis was that DWI+T2WI would have greater diagnostic accuracy in the assessment of the depth of myometrial invasion in endometrial cancer than would T2WI alone or DCE-MRI+T2WI.

## MATERIALS AND METHODS

### Patient selection

This was a retrospective, single-center study including 44 women with a median age of 68 years (range, 44-78 years). All of the women had surgically confirmed primary endometrial cancer and had undergone pelvic MRI (including T2WI, DCE-MRI, and DWI) as part of their preoperative evaluation. Patients who had undergone preoperative MRI and/or underwent surgery at other institutions were excluded. The institutional review board approved this study and waived the requirement for written informed consent.

**Figure 1:**
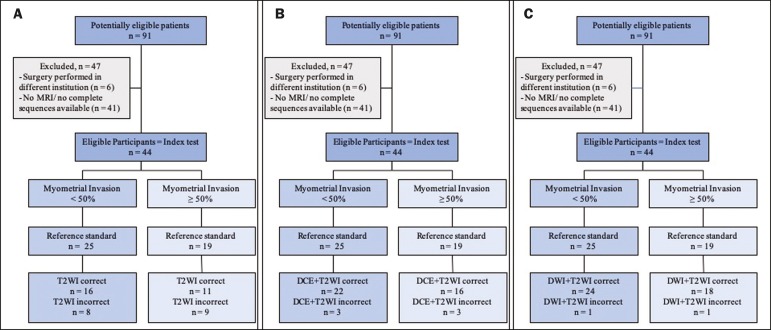
Flow of participants through the study, including the acquisition of the MRI sets T2WI (**A**), DCE+T2WI (**B**), and DWI+T2WI (**C**).

The surgical procedures were performed at a dedicated oncology center by surgeons with more than 10 years of experience in the treatment of gynecological malignancies. All of the operations were performed 3-8 weeks after the preoperative MRI examination. The result of the histopathological examination, which was performed by a pathologist who specialized in gynecology/oncology and had more than 20 years of experience, constituted the diagnostic standard for comparison. The flow of the patients through the study is depicted in Figure 1.

### MRI protocol

The MRI studies were performed in a 1.5 T MRI scanner (Intera Pulsar; Philips Medical Systems, Best, The Netherlands) with an 8-channel phased-array body coil and saturation bands (anterior and superior). Patients were asked to fast for 4 h before the examination. To reduce bowel motility and peristaltic artifacts, N-butylscopolamine bromide (20 mg) was administered via intramuscular injection before the MRI examination. During the examination, patients were placed in the supine position. 

The MRI protocol included axial imaging of the abdomen for evaluation of advanced disease, with fast spin-echo T2WI (slice thickness, 6 mm; interslice gap, 1 mm; and breath-hold), and DWI with an echo-planar technique (slice thickness, 6 mm; interslice gap, 1 mm; and b-values of 0, 500, and 1000 s/mm^2^, together with the respective ADC maps). The pelvic evaluation included fast spin-echo T1-weighted imaging obtained in the axial plane (slice thickness, 4 mm; and interslice gap, 0.4 mm) and fast spin-echo T2WI obtained in three planes-the axial plane (slice thickness, 4 mm; and interslice gap, 0.4 mm), the sagittal plane (slice thickness, 4 mm; and interslice gap, 0.4 mm), and the axial oblique plane relative to the major axis of the uterine body (slice thickness, 4 mm; and interslice gap, 0.4 mm).

For DCE-MRI, a 3D fat-suppressed gradient echo T1-weighted sequence (slice thickness, 3 mm; and interslice gap, 0.5 mm) was acquired after intravenous injection of gadopentetate dimeglumine (0.1 mmol/kg of body weight-Magnevist; Bayer HealthCare AG, Leverkusen, Germany) at a rate of 2 mL/s. Images were obtained at 0, 25, 60, 120, and 150 s in the axial oblique plane perpendicular to the major axis of the uterine body and at 240 s in the axial plane. DWI with an echo-planar technique (slice thickness, 4 mm; interslice gap, 1 mm; and b-values of 0, 600, and 1000 s/mm^2^, with the respective ADC maps) was acquired in the axial plane. When there was suspicion of cervical invasion, additional axial oblique T2WI was obtained perpendicular to the cervical canal (slice thickness, 4 mm; and interslice gap, 0.4 mm), to evaluate cervical and parametrial invasion, together with DCE-MRI, obtained at 0, 25, 60, 120, and 150 s in the sagittal plane and at 240 s in the axial oblique plane perpendicular to the cervical canal.

### Image analysis

Two radiologists specializing in urogenital radiology, with 5 and 22 years of experience, respectively, both of whom were blinded to the histopathological reports, evaluated the MRI scans and performed a consensus interpretation of the depth of myometrial invasion and stage of the cancer based on the FIGO staging system, considering three sets of sequences: T2WI, DCE-MRI+T2WI, and DWI+T2WI. In all patients, the observers started with the analysis of the T2WI sequence, evaluating the depth of myometrial invasion and the predictable stage of disease (considering cervical stromal invasion, vaginal/pelvic involvement, and lymph node or distant metastases). The observers then analyzed the DCE-MRI+T2WI sequences and evaluated the same parameters. Finally, the observers analyzed the DWI+T2WI (cognitive fusion, rather than image fusion) sequences, again evaluating the same parameters. In each case, the T1-weighted imaging was analyzed independently, to exclude potential pitfalls such as hemorrhage. The myometrial invasion was classified as superficial if the tumor had invaded < 50% of the myometrial thickness and deep if it had invaded ³ 50%.

### Histological findings

All the patients in the study sample underwent total hysterectomy, bilateral salpingo-oophorectomy, pelvic lymphadenectomy, and peritoneal lavage. The histopathological evaluation of the tumor included histological type, tumor grade, and depth of myometrial invasion (superficial or deep). The presence of cervical stromal invasion, extension to the serosa or other organs (e.g., ovaries, fallopian tubes, and peritoneum), and lymph node metastases were also evaluated.

To determine the depth of myometrial invasion, we considered two criteria. First, we calculated the total thickness of the myometrium, considering the areas that were not invaded, and deep invasion was defined as invasion of half or more than half of that thickness. Second, we determined whether tumor cells had reached the vascular plexus that separates the two layers.

### Statistical analysis

All statistical analyses were performed using R software, version 3.3.3 (a free software environment available at https://www.r-project.org). Data are presented as absolute and relative values or as proportions and 95% confidence intervals (95% CI). The diagnostic accuracy, sensitivity, specificity, positive predictive value (PPV), and negative predictive value (NPV) were calculated for T2WI, DCE+T2WI, and DWI+T2WI. The accuracy of the different sets of sequences in the evaluation of deep myometrial invasion was compared with *p*-value adjustment by the Benjamini-Hochberg procedure for controlling the false discovery rate in multiple comparisons. A similar procedure was performed to compare the accuracy in staging. Values of *p* < 0.05 were considered statistically significant.

## RESULTS

The demographic characteristics of the patients and the postoperative histological findings are summarized in Table 1. Of the 44 patients, 25 (57%) had superficial myometrial invasion, and 19 (43%) had deep myometrial invasion. Cervical invasion was observed in two patients with superficial myometrial invasion and in four patients with deep myometrial invasion, whereas serosal invasion was observed in only one patient (with deep myometrial invasion).

**Table 1:** Characteristics of and surgical findings in a sample of patients with endometrial cancer.

Variable	(N = 44)
Age at diagnosis (years), median (range)	68 (44-78)
Histological subtype, n (%)	
Endometrioid carcinoma	27 (61)
Serous carcinoma	8 (18)
Mucinous carcinoma	4 (9)
Clear cell carcinoma	3 (7)
Mixed cell carcinoma	2 (5)
Myometrial invasion, n (%)	
Superficial (< 50%)	25 (57)
Deep (≥ 50%)	19 (43)
Tumor grade, n (%)	
1	9 (20)
2	14 (32)
3	21 (48)
FIGO stage, n (%)	
IA	23 (52)
IB	14 (32)
II	6 (14)
IIIA	1 (2)

As can be seen in Table 2, the depth of myometrial invasion was correctly determined with T2WI in 27 (61%) of the 44 patients evaluated, with DCE-MRI+T2WI in 38 (86%), and with DWI+T2WI in 42 (95%). In addition, correct staging was achieved with T2WI in 22 patients (50%), with DCE-MRI+T2WI in 34 (77%), and with DWI+T2WI in 41 (93%). DWI+T2WI demonstrated higher diagnostic accuracy, sensitivity, specificity, PPV, and NPV than did T2WI and DCE-MRI+T2WI in the assessment of the depth of myometrial invasion (Table 3).

**Table 2:** Correct identification of the degree of myometrial invasion, by MRI sequence set, together with a comparison between the MRI findings and pathologic findings in terms of staging, in patients with endometrial cancer.

	Myometrial		Myometrial	
	invasion < 50%		invasion ≥ 50%	
MRI sequence set	(n = 25)		(n = 19)	
T2WI, n (%)	16 (64)		11 (61)	
DCE-MRI+T2WI, n (%)	22 (88)		16 (84)	
DWI+T2WI, n (%)	24 (96)		18 (95)	
	Pathologic stage (n = 44)			
MRI sequence set	IA	IB	II	IIIA
T2WI, n				
IA	14	7	3	0
IB	9	7	1	1
II	0	0	1	0
IIIA	0	0	1	0
DCE-MRI+T2WI, n				
IA	20	2	3	0
IB	3	12	1	0
II	0	0	2	1
IIIA	0	0	0	0
DWI+T2WI, n				
IA	22	1	1	0
IB	1	13	0	0
II	0	0	5	0
IIIA	0	0	0	1
Total	23	14	6	1

**Table 3:** Diagnostic performance of T2WI, DCE-MRI+T2WI, and DWI+T2WI in the assessment of the depth of myometrial invasion in patients with endometrial cancer.

MRI sequence set	Accuracy	Sensitivity	Specificity	PPV	NPV
Myometrial invasion ≥ 50% (n = 19)					
T2WI, % (95% CI)	61 (45-79)	58 (33-80)	64 (43-82)	55 (32-77)	55 (32-77)
DCE-MRI+T2WI, % (95% CI)	86 (73-95)	84 (60-97)	88 (69-97)	88 (60-97)	88 (60-97)
DWI+T2WI, % (95% CI)	95 (85-99)	95 (74-100)	96 (80-100)	95 (74-100)	95 (74-100)
Myometrial invasion < 50% (n = 25)					
T2WI, % (95% CI)	61 (45-76)	64 (43-82)	58 (33-80)	67 (45-84)	55 (32-77)
DCE-MRI+T2WI, % (95% CI)	86 (73-95)	88 (69-97)	84 (60-97)	88 (69-97)	84 (60-97)
DWI+T2WI, % (95% CI)	95 (85-99)	96 (80-100)	95 (74-100)	96 (80-100)	95 (74-100)

Comparing each set of sequences (Table 4), we found that, for myometrial invasion and staging, DWI+T2WI and DCE-MRI+T2WI both demonstrated diagnostic accuracy superior to that of T2WI alone, the differences being statistically significant (*p* < 0.05 for all). DWI+T2WI showed greater diagnostic accuracy than did DCE-MRI+T2WI, for myometrial invasion and for staging, although the differences were not statistically significant (*p* > 0.05 for both).

**Table 4:** Comparison between the MRI sequence sets, in terms of their accuracy in determining the depth of myometrial invasion and the pathological stage, in the preoperative assessment of endometrial cancer.

	Depth of myometrial	
	invasion	Pathological stage
MRI sequence set pair	*p*-value*	*p*-value*
T2WI vs. DCE-MRI+T2WI	0.0305	0.0300
T2WI vs. DWI+T2WI	0.0009	< 0.0001
DCE-MRI+T2WI vs. DWI+T2WI	0.2660	0.0710

* Adjusted with the Benjamini-Hochberg procedure for controlling the false discovery rate in multiple comparisons.

## DISCUSSION

**Figure 2:**
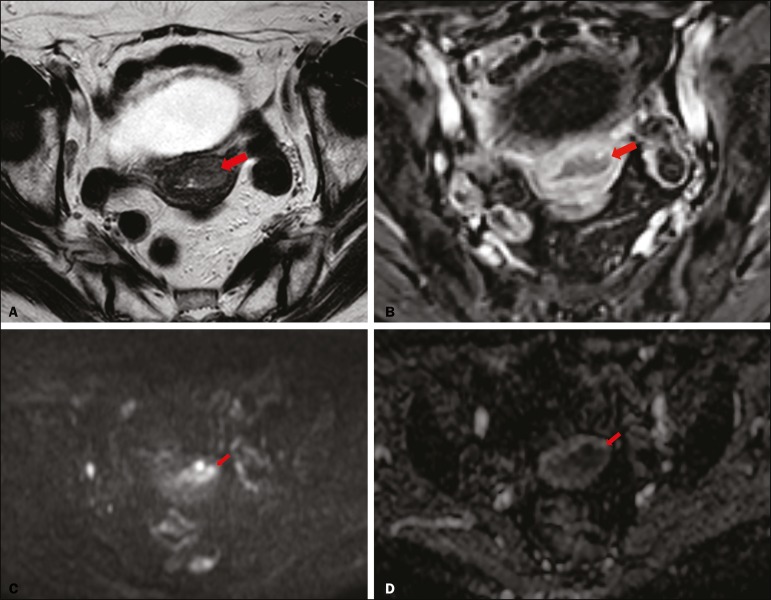
MRI of a 78-year-old woman with endometrial cancer. **A:** Axial oblique T2WI, perpendicular to the main axis of the uterus, showing an endometrial tumor that the observers judged to be invading the outer half of the myometrium (arrow). **B:** Axial oblique DCE-MRI sequence showing an endometrial tumor (arrow) with signal intensity that was low in comparison with that of the myometrium, classified by the observers as superficial myometrial invasion. **C,D:** Axial DWI sequence showing an endometrial tumor (arrows) with high signal intensity at a high (1000 s/mm2) b-value (**C**) and low signal intensity on the ADC map (**D**), classified by the observers as superficial invasion. The postoperative histological findings confirmed the superficial myometrial invasion (stage IA).

**Figure 3:**
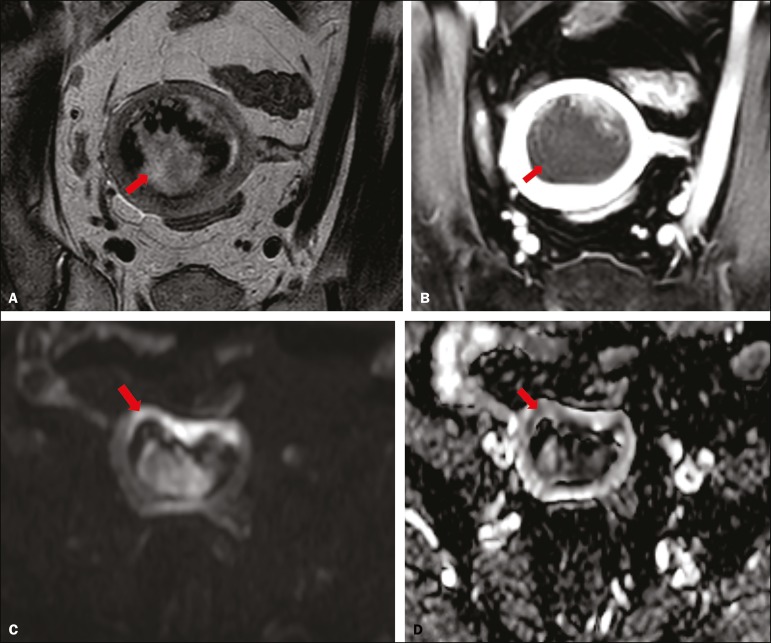
MRI of a 66-year-old woman with endometrial cancer. **A:** Axial oblique T2WI, perpendicular to the main axis of the uterus, showing a large endometrial tumor that the observers judged to present superficial myometrial invasion (arrow). **B:** Axial oblique DCE-MRI sequence (at 120 s), perpendicular to the main axis of the uterus, showing a large endometrial tumor (arrow) with a hypointense signal (in contrast with the hyperintense signal of the myometrium), classified by the observers as superficial myometrial invasion (**C**). On axial DWI, the observer consensus was that the tumor (arrows) had invaded the outer half of the myometrium, well depicted at a b-value of 1000 s/mm2 (**C**) and on the ADC map (**D**). The postoperative histological analysis confirmed the deep myometrial invasion (stage IB).

Our study confirmed that MRI is a powerful tool for the preoperative evaluation of endometrial cancer, particularly for the assessment of the depth of myometrial invasion, one of the most important prognostic factors associated with endometrial cancer. The interpretation of the diagnostic performance of DWI+T2WI and DCE-MRI+T2WI showed that both were superior to T2WI alone in the assessment of the depth of myometrial invasion and staging (Figures 2 and 3). When comparing the functional sequences (DWI and DCE-MRI), we found that DWI combined with T2WI performed slightly better than did DCE-MRI combined with T2WI, which implies potential advantages of the former combination, because DWI does not involve intravenous contrast administration and has shorter acquisition times^(^^2^^,^^17^^)^.

Recent advances in DWI hardware and acquisition contributed to the minimization of artifacts associated with arterial pulsation, peristalsis, and susceptibility effects^(^^12^^)^. Nevertheless, DWI is a motion-sensitive sequence and is susceptible to local field heterogeneity, high b-value images typically having a low degree of anatomical detail^(^^6^^)^. Therefore, DWI should be interpreted in conjunction with anatomical sequences for spatial reference and to avoid confusion with other structures and pathologies that show high signal intensity on DWI, including bowel loops, lymph nodes, endometriosis, and hemorrhagic cysts^(^^4^^,^^18^^)^. In our study, DWI in conjunction with morphologic T2WI demonstrated greater diagnostic accuracy, sensibility, specificity, PPV, and NPV in the assessment of the depth of myometrial invasion than did DCE-MRI+T2WI or T2WI alone. These results are consistent with those of the prospective study conducted by Rechichi et al.^(^^4^^)^, and those of the retrospective study conducted by Beddy et al.^(^^11^^)^, both of whom concluded that the combination of DWI and T2WI is highly accurate in assessing the depth of myometrial invasion and might be able to replace DCE-MRI in the preoperative evaluation of endometrial cancer. Our findings are also supported by those of the study conducted by Bonatti et al.^(^^19^^)^, who drew comparisons between DWI+T2WI and contrast-enhanced T1-weighted images, also showing that the former was superior. In the present study, DWI+T2WI demonstrated high sensitivity and specificity for detecting deep myometrial invasion, with an NPV of 96%, suggesting that a negative DWI result can reliably rule out deep myometrial invasion, which is in keeping with the findings of the meta-analysis performed by Das et al.^(^^20^^)^. Nevertheless, when we compared the accuracy of DWI+T2WI with that of DCE-MRI+T2WI, the differences were not statistically significant, which is consistent with the results obtained in the meta-analyses performed by Andreano et al.^(^^2^^)^ and Deng et al.^(^^21^^)^.

Morphologic evaluation with T2WI provided a high degree of anatomical detail to assess the uterus and the pelvis but was found to be limited in the assessment of myometrial invasion. On T2WI, endometrial cancer in the endometrial cavity usually has a signal intensity higher than that of the junctional zone, although confounding factors, such as an unclear junctional zone (common in postmenopausal women) and poor tumor-to-myometrium contrast, as well as myometrial compression by polypoid tumors, leiomyomas, or adenomyosis, reduce the accuracy of the technique^(^^4^^,^^11^^,^^20^^)^. In our study, T2WI correctly assessed the depth of myometrial invasion in 27 (64%) of the 44 patients evaluated, demonstrating lower specificity than in previous studies^(^^4^^,^^19^^)^. The anatomical detail provided by T2WI is also important for the assessment of lymph node metastases^(^^3^^,^^20^^)^. Because of their high signal intensity at high b-values, lymph nodes are easier to identify on DWI than on T2WI. However, DWI is still limited in predicting lymph node metastases and the DWI findings should be considered together with the classical morphological criteria^(^^6^^)^: short axis diameter > 8 mm for pelvic lymph nodes and > 10 mm for abdominal lymph nodes; irregular contours; necrosis; and clusters of lymph nodes.

Our results suggest that DWI+T2WI is superior to DCE-MRI+T2WI in the assessment of the depth of myometrial invasion, DWI+T2WI having a diagnostic accuracy of 95%, compared with only 86% for DCE-MRI+T2WI. On DCE-MRI, endometrial tumors enhance at ? 30 s (in the arterial phase, earlier than does normal endometrium), which allows the detection of small tumors confined to the endometrial cavity, as does the fact that most tumors are hypovascular compared with normal myometrium^(^^22^^)^. Maximum contrast between the high signal intensity of normal myometrium and the low signal intensity of endometrial cancer occurs 120-180 s after contrast administration, in the equilibrium phase^(^^17^^)^. However, some endometrial tumors are either isovascular or hypervascular in comparison with the myometrium, which hinders their evaluation^(^^23^^)^. In addition, adenomyosis, tumor extension to one or both of the cornua, loss of the junctional zone, and peritumoral inflammatory enhancement have been shown to reduce the accuracy of DCE-MRI^(^^11^^,^^19^^)^. Our study also showed that T2WI and DCE-MRI+T2WI are both less accurate than is DWI+T2WI in the staging of endometrial cancer. The proportion of correctly staged patients increased from 77% (n = 34) with DCE-MRI+T2WI to 93% (n = 41) with DWI+T2WI. However, our sample included a small number of patients with advanced-stage disease (only six of the patients had cervical stromal invasion and none had abnormal lymph nodes), which limits the comparative evaluation of the staging. On DWI, restricted diffusion disrupting the cervical stroma is consistent with cervical stroma invasion, which is also associated with lymph node metastases and poor survival^(^^10^^,^^24^^)^. MRI is also helpful in diagnosing advanced disease involving the adnexa and the peritoneum, which usually contraindicates laparoscopic and robotic surgery^(^^25^^)^. Our results are consistent with those presented by Beddy et al.^(^^11^^)^, differing only in that, in our study, more tumors were understaged than were overstaged, with DCE-MRI-T2WI (7 vs. 3) and with DWI-T2WI (2 vs. 1). However, because of the retrospective nature of our study, it was not possible to assess the real effects that understaging and overstaging had on patient care. Nevertheless, the high accuracy of DWI+T2WI in the assessment of FIGO staging, as demonstrated in the present study, could contribute to better preoperative selection of patients for appropriate therapy.

We recognize that our study has certain limitations. First, because it was a retrospective study, we could not assess exposures or outcomes. However, our dataset featured accurate recordkeeping and a satisfactory temporal relationship between preoperative MRI assessment, surgery, and histopathological reporting. Second, our sample size was small (n = 44), which precluded the investigation of other potential causes of heterogeneity, such as the real impact of pitfalls and confounding factors related to accurate estimation of the depth of myometrial invasion. Another important limitation is the fact that the analysis of the different sets of sequences was not randomized-all T2WI sequences being interpreted first, followed by the DCE-MRI+T2WI sets and finally by the DWI+T2WI sets-which could have introduced a learning bias. In addition, the level of interobserver agreement was not assessed and all of the studies were evaluated by two radiologists specialized in urogenital radiology, which could limit the generalizability of the results. Finally, the MRI studies were conducted during a period when DWI was performed only in the axial plane, whereas DCE-MRI was performed in two planes, including one axial oblique plane perpendicular to the major axis of the uterine body. Nevertheless, our results show that DWI+T2WI was the superior protocol.

## CONCLUSION

Our study confirmed the high diagnostic performance of MRI in the preoperative assessment of endometrial cancer. The combination of DWI and morphological T2WI demonstrated superior diagnostic accuracy in the assessment of the depth of myometrial invasion when compared with that of DCE-MRI and T2WI, indicating that DWI+T2WI is a potential replacement for DCE-MRI in the preoperative staging of endometrial cancer, especially for patients in whom contrast agents are contraindicated.
